# Reduce oral health anesthetic emissions for the sake of health

**DOI:** 10.3389/fdmed.2026.1773618

**Published:** 2026-03-23

**Authors:** M. Nevison, M. Monopoli, M. Mutis, P. Gudsoorkar, S. Mehta

**Affiliations:** 1 Health Sciences, McMaster University, Hamilton, ON, Canada; 2 Solidarity Dental Foundation, Cincinnati, OH, United States; 3 American Association of Hospital Dentistry, Grapevine, TX, United States; 4 Department of Environmental & Public Health Sciences, University of Cincinnati, Cincinnati, OH, United States

**Keywords:** anesthesia environmental impact, environmentally sustainable oral healthcare, global health, oral health emissions, oral health guidelines

## 1 Introduction

The climate crisis continues to devastate global health, with the World Health Organization naming global warming as the largest global threat to health in the twenty-first century, and this emphasizes the increasing importance of sustainability within oral health (1, 2). Impacts from the climate crisis on human health are experienced on a global level from heat exposure, changes to infectious disease patterns, changes in migration, extreme weather, harming food security, and include both physical and mental health effects, making this intersection a responsibility for health professionals (1–4). This article proposes that reducing dental anesthetic emissions is an actionable component of oral health professionals’ responsibility to global health and equity. We synthesize selected recent guidance and emissions metrics to identify practical, implementable shifts in dental sedation practice.

This article contextualizes oral health professionals’ responsibility to health and equity through the need to improve sustainable practice specifically regarding evidence-based anesthetic emissions guidelines. Oral health professionals are critical to healthcare sustainability, as healthcare in general is resource-intensive contributing 4.4–5.2 percent (%) global greenhouse gas (GHG) emissions (5). Further, the focus on anesthetics has shifted from being ignored based on the idea of medical necessity to a more addressable factor compared to other health care sources like transportation or energy use (6). Oral health professionals are relevant in discussions of anesthetics and environmental health due to current reliance on nitrous oxide (N_2_O) and other inhalation anesthetic gases within dental settings and procedures (2, 3, 12). Environmental impacts are not experienced equally across or within nations, reinforcing the responsibility to global health equity (3). The intersection of environmental and oral health extends to anesthetic emissions making their reduction a matter of global equity.

### 1.1 Headings

#### 1.1.1 Comparing oral health anesthetic emissions

Current practice includes use of inhaled anesthetic gases in oral health sedation, which contributes to the production of GHG emissions (1–14). Greenhouse gases are a significant focus of environmental sustainability due to their role in global warming as they reflect and trap infrared radiation in the atmosphere (4–7, 10, 13, 14). Commonly used gases are identified in Table 1, and are all GHGs that impact global warming (1, 4, 7, 8, 11, 12).

**Table 1:** Key evidence of oral health anesthetic emissions and global health responsibility.

Key categories	Key evidence
Emissions	● climate crisis impact on global health: ○ physical and mental effects ○ infectious disease patterns ○ extreme weather ○ food security (1–4) ● inequitable health consequences within and between countries (3) ● greenhouse gases drive warming by trapping infrared radiation in the atmosphere (4–7, 10, 13, 14).
Anesthetic emissions	● Current inhalation anesthetic gases relied on by oral health professions (desflurane, N_2_O, sevoflurane, isoflurane) (2, 3, 12). ○ greenhouse gases that impact global warming (1, 4, 7, 8, 11, 12) ○ N_2_O also depletes ozone, (1, 4, 6, 8, 13). ● considering the GWP_100_: ○ desflurane had the highest impact (2,540) ○ isoflurane (510) ○ N_2_O (289) ○ sevoflurane (130) (7) ● chronic N_2_O exposure poses employee safety risks (3)
Oral health guidelines	● avoid N_2_O and desflurane (2–6, 11, 12, 15) ● transition to intravenous, local, or regional anesthesia (2–6, 11, 12, 15) ● replace central piping to canisters (12) ● explore scavenging technology (2–6, 11, 12, 15)
Oral health transitioning	● resource guides and implementation tools exist (12, 15) ● professional environmental awareness has increased (3, 11) ● healthcare transitioning efforts are underway, including in hospitals (11, 12) ● barriers: patient outcomes, costs, efficiency, anesthesiologist resistance to practice change, limited environmental health awareness, and limited leadership interest (11, 12)

Significantly, desflurane has a worse global warming potential over 100 years (GWP_100_) than carbon dioxide (CO_2_) (5, 7). Per-molecule, desflurane is more effective at absorbing infrared radiation than CO_2_ (7). Professionals should be mindful of the potential GHG emission harm, as ranked in Table 1 by GWP_100_ (12). Desflurane has a 100-year CO_2_ equivalent impact of approximately 50 times greater than sevoflurane, highlighting the urgency of revising anesthetic gas practices, especially given existing institutional transitions (7, 9, 12). Further, N_2_O is not only a GHG, but is also an ozone depleter making its impact significant to climate change, contextualizing recommendations to reduce its use (1, 4, 6, 8, 13). Reducing emissions should be viewed in perspective of impact and global anthropogenic CO_2_ emissions, considering this, anesthetic emissions are a critical target to reduce environmental impacts (4, 8, 13).

The use of GWP_100_ as a measurement is not without critique, as Slingo & Slingo (2024) include that the use of GWP as a comparative to CO_2_ does not reflect the complexity of atmospheric regulation and varying atmospheric concentrations. Instead, recommending using radiative forcing as a measure, as this would measure concentrations rather than the CO_2_ equivalent (13). Slingo & Slingo (2024) note that N_2_O is a GHG but also an ozone-depleting substance with a long lifetime of 123 years, with an abundance in the atmosphere, agreeing with GWP_100_-based literature regarding recommendations to reduce N_2_O use where possible and to use low fresh gas flows to reduce the environmental harm and improve population health through evidence-based sustainable practices.

#### 1.1.2 Current guidelines and critiques

Current guidelines, as shown in Table 1, include recommendations to avoid N_2_O and desflurane, transition to intravenous or local regional anesthesia to bypass inhalation, and to explore scavenging technologies to reduce waste (2–6, 11, 12, 15). When comparing oral health anesthetic emissions, recommendations to reduce N_2_O and desflurane are based on reducing the release of emissions to mitigate the environmental impact while ensuring patient safety and treatment quality (12). Further supporting this mitigation guideline, lower-emission alternatives such as sevoflurane and isoflurane are already in use in general anesthesia (12). Mitigation guidelines prioritize N_2_O and desflurane, making this change an important call to action for oral health professionals to implement (12).

Current guidelines also suggest bypassing inhalation and transitioning to intravenous or local regional anesthesia to further reduce emissions waste within healthcare when appropriate for treatment and patient outcomes (12). There are specific recommendations to transition away from central piping systems for N_2_O, as these systems result in continuous leaks into the environment and substantial loss of N_2_O before delivery (12). Instead, if N_2_O is to be used, current guidelines suggest using portable canisters to reduce leakage (12). There has been interest in scavenging technology to mitigate the impact of inhaled anesthetic waste by potentially capturing or destroying the inhaled anesthetic before it reaches the environment (12). Table 1 included the limited impact research on scavenging mitigation and therefore is not considered a priority at this time due to other more readily implementable guidelines described (12). Scavenging devices and reducing the use of inhaled anesthetics may also be important for employee safety due to leakage and chronic exposure, making these sustainable changes further relevant to practitioners (3, 5). Chronic exposure to N_2_O has been attributed to adverse health effects, so improving scavenging to reduce ambient exposure or transitioning to intravenous anesthetics may support practitioner health (3). The downstream treatment approach in dentistry, including the use of anesthetic gases, is concerning considering the global burden like increasing incidence of caries in permanent teeth of children (16). An upstream strategy of incidence prevention is important to reduce treatment demand and associated anesthetic emissions (17).

#### 1.1.3 Transition in practice – examples

Efforts to reduce anesthetic emissions have already begun, and there are tools to help workplaces transition, including guides from the Institute for Healthcare Improvement (2024) and the American Society of Anesthesiologists (2022) that utilize tool templates to aid in strategies to implement mitigation goals. Further, awareness about how environmental health impacts oral health has been increasing, indicating an understanding by oral health professionals that minimizing the environmental harm of current practices is part of equitable global health, but there is still resistance from some practitioners to transition (3, 11).

Table 1 includes some common concerns around transitioning current oral health anesthetic practice towards more sustainable alternatives, for example, patient outcomes, making evidence-based guidelines important (11, 12). When working with health organizations in British Columbia, Canada, it was found that the health organization Providence Health Care saw cost savings from reduced desflurane use with 78%–88% of CO_2_ equivalent also reduced (11). Some challenges noted was a lack of willingness from anesthesiologists to alter practices, questions if sevoflurane was equivalent to desflurane in terms of anesthetic use, lacking equipment to change practice, concerns of costs, lack of awareness of environmental harm, and lack of interest from leadership to implement emission reduction initiatives (11). Despite these barriers, all participants interviewed supported banning and removing desflurane as well as using low fresh gas flow rates with sevoflurane as a transition towards intravenous anesthesia to support environmental and human health (11). Importantly, despite concerns regarding patient outcomes, cost, and treatment efficacy, the connection between environmental and human health was generally supported.

#### 1.1.4 Global health and equity context

As mentioned, environmental health and oral health are interconnected, environmental harm contributed by oral health practices have implications on global health. Hackley & Luca (2024) note that environmental health remains to be an equity issue. The impacts from emissions do not solely impact those who release them, but rather those who contribute the least to GHG emissions are most vulnerable to the consequences due to less resources to adapt to and mitigate risks (3). This is a global pattern of inequity experienced between countries, but it also exists within countries (3). For example, in the United States (US), vulnerability to climate change is experienced disproportionately by low-income, communities of color, immigrant, Indigenous, children, and those with disabilities, thereby connecting how social determinants impact the disease burden (3). Further, Jones et al. (2024) note ‘first do no harm’ in reference to the importance for health professionals to weigh the potential harm over the benefits of interventions, which encompasses anesthetic practice and environmental health. Environmental health remains an unprecedented global health concern which requires interdisciplinary efforts to not just address but to prevent factors of health inequity making anesthetic sustainability a critical next step for oral health.

## 2 Discussion

Oral health professionals have a responsibility to push for sustainable progress to address global health and equity outcomes. Environmental health is interconnected with human health, and climate change requires prioritization and changes in policy to ensure a sustainable future, including within the oral health anesthetics field (1, 2, 12). Oral health professionals have a responsibility to health, and this includes prioritizing environmental health by advocating for and implementing evidence-based guidelines to reduce the environmental impact of current anesthetic practice. While barriers exist, oral health practitioners play an important role in researching and engaging in actionable guidelines to ensure sustainable dentistry and address the growing harm of environmental destruction to global health. Oral health professionals have a fundamental ethical responsibility to ‘first do no harm,’ which includes the intersection of environment and human health. Bringing sustainability to the forefront of oral health anesthetic use will support global goals to improve global health outcomes by reducing the field's contribution to environmental harm and corresponding inequitable health consequences. Health equity and the environment are interconnected, meaning environmental health is oral health, so sustainable practice is pivotal.

Emphasizing the responsibility oral health professionals have to health with the interconnection of environmental health would build global health and equity capacity within oral health. Reducing the environmental harm of oral health anesthetic practice while maintaining patient outcomes is critical for equitable global health. There are already guidelines to support oral health professionals in transitioning towards less environmentally harmful anesthetics and alternative delivery methods, making this an implementable call to action for professionals. We encourage oral health professionals to support the adoption of sustainable practice, push policy levers for evidence-based change, and advocate for sustainable oral health practice for the health of not just current but future patients around the world. For the sake of health, oral health professionals must not just support, but prioritize, evidence-based sustainability guidelines.

Dental anesthetic emissions are a feasible, near-term target for decarbonizing oral healthcare without compromising patient safety. In routine dental settings, priority actions include: (1) reducing N_2_O use when clinically appropriate and strengthening alternatives, (2) addressing N_2_O delivery losses by avoiding high-leak systems (e.g., minimizing central piping where feasible), and (3) adopting evidence-based guidance that aligns emissions reduction with occupational safety. Future work should quantify dental-setting–specific emissions and implementation outcomes (patient experience, cost, and equity effects) to guide scalable practice change.
